# Exploring Social Sharing Value: Effects on Customer Attitudes and Behaviors in Restaurant Livestreaming

**DOI:** 10.3390/bs14070621

**Published:** 2024-07-21

**Authors:** Zihan Yang, Vincenzo Liu, Chan Lyu

**Affiliations:** School of Business, Macau University of Science and Technology, Macau, China; 2009853dbm30001@student.must.edu.mo

**Keywords:** social sharing value, trust, satisfaction, word of mouth, behavior intention, livestreaming, restaurant

## Abstract

In China, the integration of livestreaming into restaurant marketing has transitioned from mere entertainment to a vital business tool. This study examines the influence of social sharing value (SSV) on customer attitudes and behavioral intentions within the context of restaurant livestreams, applying the stimulus-organism-behavior-consequences (SOBC) model. Analyzing data from 1139 livestream viewers using partial least squares-path modeling (PLS-PM), the results reveal that SSV significantly enhances viewers’ trust, satisfaction, word of mouth (WOM), and behavioral intentions. Trust was shown to elevate satisfaction, which, in turn, positively impacts WOM and purchase intentions. Among the SSV’s components, brand intimacy emerged as highly influential. Notably, behavioral intention was found to significantly influence WOM activities, underscoring the critical role of proactive customer behaviors in promoting the brand. This study extends social exchange theory by quantifying relationship quality and adapting it to digital consumer interactions in the restaurant industry. The findings highlight the importance of cultivating SSV to bolster customer trust and satisfaction, thereby enhancing loyalty and advocacy. Effective engagement through livestreaming can amplify brand intimacy, establishing it as an indispensable strategy for maintaining competitiveness in the restaurant sector.

## 1. Introduction

The sharing of information by restaurants through social media platforms has become an essential marketing tool. Various channels, such as Weibo, WeChat groups, subscription articles, live commerce, and especially livestreaming, are increasingly utilized by restaurants to attract customers. These platforms provide customers with access to food and related information, including ingredients, production processes, sales status, food tasting experiences, and the cultural and historical context of the restaurant. Restaurants often design games or promotional activities to engage customers and enhance their experience.

Traditionally, the catering industry has focused on enhancing dine-in services, which significantly differ from the dynamic digital interactions facilitated by livestreaming. Livestreaming allows guests to virtually experience the restaurant’s atmosphere and observe the food production process, thereby eliciting stronger emotional responses and engagement. For instance, Luo Min’s 19-hour TikTok livestream featuring pre-made dishes attracted 95.87 million viewers, resulted in daily sales of 9.56 million units, and gained 3.97 million followers in a single day, demonstrating the profound impact of well-executed livestreams [1].

Distinct from traditional e-commerce, livestreaming provides a more “natural” shopping experience that mimics returning to physical stores. This approach not only enhances visual appeal but also aligns product presentations with user needs, as demonstrated by Herborist’s TikTok broadcasts. These streams effectively combined product showcases with thematic content, attracting significant viewership. Additionally, strategic partnerships, such as the one between Meituan and Kwai, which launched a direct purchasing feature through livestreams, exemplify how platforms are evolving to integrate more seamless shopping experiences directly into livestreams [2,3,4,5].

While livestreaming has become a popular e-commerce method, research on the impact of social media sharing and livestreaming on customers’ attitudes and purchase intentions remains limited [6]. Existing studies on livestreaming commerce are still in their early stages and primarily focus on three key areas: the motivations of users shopping through livestreaming, the current status of user participation, and the consumers’ perceived value of livestreaming influenced by trust and engagement [7]. Visual and voice guidance in live shopping are also identified as crucial factors influencing purchasing intentions [8]. Furthermore, some research utilizes data from live broadcast platforms to inform sellers’ marketing strategies and tactics on platforms such as Facebook [9].

Despite the accelerating integration of digital strategies in the restaurant industry, the intricate influence of social sharing value (SSV) on customer attitudes and behaviors via livestreaming is not well documented. SSV encompasses critical dimensions such as brand intimacy, engagement, individual recognition, and community belonging—all pivotal in forging resilient customer relationships and reinforcing brand loyalty within the digital domain. Traditionally, the bulk of research has centered on the broad impacts of direct marketing and general social media advertising, often neglecting the deeper, behavior-shaping capacities of livestreaming. This oversight persists despite growing evidence that livestreaming can uniquely enhance customer engagement and influence through real-time, interactive experiences that traditional media channels cannot offer.

Most existing studies have not adequately addressed how specific elements of SSV, such as brand intimacy—which involves personalizing customer interactions to foster a closer connection with the brand—and community belonging—where customers feel part of a brand’s community through shared experiences and values—manifest and impact consumer behavior in livestream settings. Moreover, while many studies explore general digital marketing effects, few dissect the nuanced psychological and behavioral outcomes specific to livestreaming in the restaurant industry, such as how livestreaming can modify customer expectations or satisfaction during real-time interactions.

This study aims to bridge these gaps by specifically investigating how the various facets of SSV influence key customer attitudes and behaviors, such as trust in the brand, satisfaction with the service, and the likelihood of recommending the restaurant to others. By focusing on the restaurant sector, which has traditionally relied on physical ambiance and service quality to attract and retain customers, this research provides new insights into how digital transformations such as livestreaming are reshaping industry paradigms. This approach not only differentiates from previous studies by delving into the specific impacts of livestreaming within the SSV framework but also highlights its implications for customer relationship management in an increasingly digital marketplace.

In doing so, this research contributes to a more comprehensive understanding of digital consumer behavior, particularly in how livestreaming as a form of social media interaction can extend beyond mere promotional activity to significantly affect how customers perceive and interact with brands in the restaurant industry. This is crucial for developing more effective digital marketing strategies that harness the full potential of SSV to enhance customer engagement and loyalty.

This study aims to bridge the research gap by employing the stimulus-organism-behavior-consequences (SOBC) model to systematically analyze how livestreaming affects key consumer metrics such as trust, satisfaction, word of mouth, and behavioral intentions [7]. As digital platforms rapidly transform the marketing landscape, particularly in the restaurant industry, SSV plays a crucial role in shaping customer perceptions and behaviors. Despite its transformative potential, the specific impacts of livestreaming—a prominent component of digital marketing—on customer attitudes and behaviors within independent restaurants remain poorly understood.

The primary objective of this research is to explore the influence of livestreaming on customer interactions and their subsequent effects on brand perception and customer loyalty. This involves examining components such as brand intimacy, engagement, individual recognition, and community belonging, as well as their role in enhancing customer trust, satisfaction, and loyalty. By utilizing the SOBC model, the study aims to delineate the causal relationships between the livestreaming stimuli and customer responses in a restaurant setting and extend this analysis to the consequences of these behaviors. This model is particularly suited to the livestreaming context, where the immediate interactive nature of the medium can lead to direct and measurable impacts on consumer behavior and subsequent business outcomes.

The SOBC model provides a thorough framework that not only captures the initial responses (organism) to stimuli but also extends to encompass the resultant behaviors and their enduring impacts, such as the transition from behavioral intentions (BI) to word of mouth (WOM). This holistic approach allows for a more detailed analysis of how initial interactions lead to specific consumer behaviors—such as the intention to recommend or repurchase—which subsequently manifest as word-of-mouth endorsements. These behavioral outputs, driven by strong intentions, typically translate into impactful consequences, amplifying the brand’s reputation and reach through customer advocacy. Thus, understanding this progression from BI to WOM is crucial for businesses aiming to leverage consumer behaviors for long-term benefits. This approach allows for a deeper understanding of how engagements through livestreaming can lead to sustained customer relationships and loyalty, which are critical for business success in the competitive restaurant industry. By mapping out these extended interactions, the research will provide insights into the efficacy of livestreaming strategies and their potential to foster lasting engagement and loyalty among viewers.

Incorporating the SOBC model enriches the analysis by capturing the feedback loop where consumer behaviors lead to tangible outcomes that, in turn, influence future consumer interactions and business strategies. This holistic approach is crucial for understanding the dynamics of digital marketing in the restaurant sector and for crafting strategies that leverage livestreaming to enhance customer engagement and achieve business growth. This study will not only fill the existing research void but also offer practical guidance for leveraging digital innovations to enhance customer value and business performance in the hospitality industry.

While the impact of social media marketing is well-documented, the specific dynamics of livestreaming as a distinct marketing tool have received limited attention, particularly in the context of independent restaurants. Previous studies have focused on broader digital marketing strategies or the effects of social media platforms such as Facebook and Instagram, often overlooking the nuanced interaction dynamics and real-time engagement that livestreaming facilitates. Understanding the impact of livestreaming on customer attitudes and behaviors is crucial for restaurant owners and marketers, as it can significantly enhance customer engagement and loyalty—key drivers of business success in the competitive restaurant industry. This research will provide valuable insights into effective livestreaming strategies that can maximize social sharing value, thereby improving customer retention and attracting new patrons through enhanced digital interaction quality. By addressing these objectives and filling the identified research gaps, this study will contribute to the broader discourse on digital marketing’s impact in the hospitality industry, offering actionable strategies that can be employed to leverage technology for business growth and customer satisfaction. This research will not only enrich academic literature but also serve as a practical guide for restaurateurs looking to harness the power of livestreaming to enhance their marketing efforts.

## 2. Literature Review and Hypotheses

SSV is structured through four key aspects: brand intimacy, brand individual recognition, brand engagement, and brand community belonging. Social exchange theory, as proposed by the sociologist Homans [10], provides the theoretical foundation for understanding these dynamics, positing that individuals engage in social interactions with the aim of maximizing benefits and minimizing costs. This theory highlights the principle of fairness, which suggests that unreciprocated benefits can induce feelings of guilt and embarrassment, motivating individuals to reciprocate with positive behaviors in interactive relationships [11].

Despite its comprehensive application, the social exchange theory encounters limitations when applied to digital interactions, particularly in the context of livestreaming and social media. Traditional applications of the theory primarily focus on tangible exchanges, such as goods and services, and may not fully capture the nuances of digital engagements where intangible benefits, such as emotional support and social recognition, play a significant role. This gap becomes apparent in environments such as social media livestreaming, where the exchanges are less about tangible outputs and more about the relational and emotional connections that brands and consumers build.

Additionally, while the social exchange theory addresses the reciprocity and fairness in exchanges, it may not adequately account for the complexities of online communities and digital interactions. In the digital realm, the immediacy and anonymity of interactions can alter traditional social exchange dynamics, leading to new patterns of behavior that are not fully explained by traditional theories.

Furthermore, the concept of SSV in digital marketing expands upon social exchange theory by incorporating modern elements of digital interaction that influence consumer behavior. Brand intimacy, individual recognition, engagement, and community belonging each relate to how consumers perceive and interact with brands online, offering a richer array of interactions than traditional exchanges considered in social exchange theory. These elements of SSV reflect a deeper and more complex layer of social interactions facilitated by digital platforms, which challenge the conventional boundaries of the social exchange theory.

This research underscores the need to adapt and extend the social exchange theory to better understand the subtleties of social interactions in the digital age, particularly those mediated through livestreaming platforms. By focusing on SSV and its components, this study addresses the limitations of the social exchange theory in digital contexts and highlights the unique contributions of emotional and communal exchanges that drive consumer behavior in online settings.

In restaurant livestreaming, hosts engage viewers through interactive methods such as sharing, displaying, and chatting, effectively transmitting business information [12]. As recipients of these exchanges, customers often experience pleasure guided by the reciprocity principle and develop a commitment to maintain relationships with the brand, engaging in reciprocal behaviors such as trust, satisfaction, and recommendations [13,14]. This foundational theory supports examining consumer purchase behaviors in livestream contexts, highlighting the influence of reputation and reciprocity on electronic word-of-mouth behaviors.

Trust, a significant construct within the social exchange theory, is described as the desire to rely on an exchange partner in whom one has confidence [15]. Brand intimacy encompasses a long-term commitment to brands, enhancing consumers’ enjoyment of experiential benefits or interactions with the brand [16]. This leads to frequent and meaningful interactions, deepening the merchant-customer relationship. As consumers become more familiar with the brand, their loyalty and commitment deepen, enhancing their overall brand evaluation and satisfaction.

Customer participation meets higher psychological needs such as respect and self-actualization through interactions with the brand. This participation not only satisfies these needs but also promotes positive consumer behaviors, enhancing brand recognition and satisfaction [17]. Livestreaming, as a novel Internet marketing tool, emphasizes real-time interaction and high customer participation, making it particularly effective during events such as the COVID-19 pandemic, which significantly shifted social and economic structures [18].

Brand communities can act as a “safe haven” for customers by fulfilling their need for kinship [19]. Once individuals become members of such a community, they experience a heightened sense of social identity and develop more favorable self-perceptions as part of the group [20]. A sense of belonging positively influences tourists’ behavioral intentions. Companies capable of evoking a sense of belonging and enthusiasm are more successful in capturing consumers’ hearts and minds [21]. Customers who feel valued are more likely to influence the restaurant’s decisions.

This study employs the SOBC model to explore how livestreaming affects key consumer metrics such as trust, satisfaction, word of mouth, and behavioral intentions. By examining the components of brand intimacy, engagement, individual recognition, and community belonging and their role in enhancing customer trust, satisfaction, and loyalty, the study delineates the causal relationships between livestreaming stimuli and customer responses, extending this analysis to the consequences of these behaviors.

The SOBC model offers a comprehensive framework that considers not only the immediate reactions to stimuli but also the resulting behaviors and their long-term consequences. This approach allows for a deeper understanding of how engagements through livestreaming can lead to sustained customer relationships and loyalty, critical for business success in the competitive restaurant industry. By mapping out these extended interactions, the research will provide insights into the efficacy of livestreaming strategies and their potential to foster lasting engagement and loyalty among viewers.

Incorporating the SOBC model enriches the analysis by capturing the feedback loop where consumer behaviors lead to tangible outcomes that, in turn, influence future consumer interactions and business strategies. This holistic approach is crucial for understanding the dynamics of digital marketing in the restaurant sector and for crafting strategies that leverage livestreaming to enhance customer engagement and achieve business growth. This research will not only fill the existing research void but also offer practical guidance for leveraging digital innovations to enhance customer value and business performance in the hospitality industry.

Therefore, we hypothesize:

**H1.**  *Social sharing value has a positive impact on satisfaction*.

This study integrates the SOBC model to explore how livestreaming enhances trust among consumers, a critical factor for effective cooperative interactions between clients and service providers [22]. Unlike traditional online shopping, which primarily involves straightforward communication of product details via instant messaging and post-purchase evaluations, livestream shopping offers real-time, direct interactions between customers and live streamers. Immediate responses from live streamers enrich the communication experience, allowing customers to evaluate the attentiveness of the streamers and the quality of the product or service directly. Such interactions not only meet customer needs but also elevate the perceived service quality, thereby fostering trust. Livestreaming significantly enhances emotional connections between customers and brands, leading to stronger trust, which is directly linked to increased brand trust [23,24].

Within this framework, the components of SSV, such as brand intimacy, engagement, individual recognition, and community belonging, are critical. Brand intimacy enhances the closeness customers feel with a brand, often through personalized interactions during live streams. Brand engagement involves customers’ active participation and sustained attention, which solidifies their connection to the brand. Brand individual recognition occurs when customers see their unique preferences acknowledged in real time, enhancing trust. Lastly, brand community belonging, fostered through shared experiences and interactions among community members during live sessions, cements trust and loyalty toward the brand.

By leveraging customer feedback to refine offerings and maintaining effective communication, customers feel valued, which fosters stronger recognition and trust in the brand [25]. The integration of SSV elements, particularly in livestream shopping environments, plays a pivotal role in enhancing consumer trust. Therefore, the SOBC model allows us to hypothesize that SSV significantly influences consumer trust, making it a potent factor in consumer behavior in livestream contexts. This model’s comprehensive approach captures the immediate interactions (Stimulus and Organism) and extends to analyze the resultant behaviors and their long-term consequences (behavior and consequences), providing a holistic view of the impacts of digital marketing innovations in the restaurant industry. Therefore, we hypothesize:

**H2.**  *Social sharing value has a positive impact on trust*.

In the era of mobile internet, word of mouth (WOM) remains a pivotal aspect of brand marketing, defined as consumer-generated comments—both positive and negative—about products, services, or businesses experienced by customers. These comments are typically shared on platforms such as shopping websites, online forums, and virtual communities where they influence other consumers’ perceptions and behaviors [26].

The impact of word of mouth is significantly shaped by the sender-receiver dynamics, the message’s depth and potency, and the method of delivery. Intimacy, for instance, profoundly influences brand love, fostering outcomes such as brand loyalty, robust word-of-mouth advocacy, and resilience against adverse information. During interactions, when individuals receive recognition or rewards, such as coupons or discounts during a livestream, they develop psychological attachments that encourage maintaining and nurturing these beneficial relationships. This dynamic encourages sustained engagement and leads to word-of-mouth endorsements due to the perceived value of the relationship [27].

Engagement in marketing is recognized as a crucial driver for consumer decision-making regarding product selection and service provider choices. Word of mouth serves as an informal communication channel where individuals discuss their experiences with services or products [28]. There is a noted positive impact of active involvement on behaviors such as recommendations [29,30]. Through participation, customers gain control and a sense of achievement, which enhances their social identity and self-presentation, culminating in a desire to share and recommend.

Restaurants, for instance, enhance customer experience by introducing both their in-restaurant environment and dishes alongside prefabricated dishes, allowing customers to enjoy similar dining experiences at home. When customers can suggest adjustments to these dishes and see their suggestions implemented, they feel valued and are more likely to recommend the restaurant to others, thus building a positive reputation.

The SOBC model in this context analyzes how such interactions influence consumer behavior. By incorporating this model, the research aims to understand how the initial stimulus (livestreaming interactions) affects the organism (consumer’s psychological response), influences their behavior (participation and word-of-mouth activities), and leads to specific consequences (strengthened brand loyalty and community engagement).

This comprehensive approach not only captures immediate interactions but also considers the long-term consequences of these behaviors, thus providing a richer understanding of digital marketing dynamics in the restaurant sector. By exploring how these digital interactions influence consumer behavior, the study aims to offer insights into effective strategies for enhancing customer engagement and loyalty, pivotal for success in the competitive restaurant industry.

**H3.**  *Social sharing value has a positive impact on word of mouth*.

Brand intimacy is crucial in driving favorable word of mouth, enhancing loyalty and purchase intentions, and boosting brand enthusiasm, serving as a fundamental component of effective marketing strategies [31]. Its significance is particularly noted in China’s web celebrity economy, where fostering intimacy is essential for attracting and retaining followers [32]. Social media sharing allows restaurants to broadcast real-time information, providing more convenient consumption avenues, thus enhancing purchase intentions [33]. For instance, Ele.me leverages the TikTok open platform to facilitate services ranging from recommendations to instant delivery, increasing user engagement and brand intimacy, which in turn encourages more frequent consumption.

Positive shopping experiences and robust brand engagement significantly elevate consumers’ propensity to purchase. The perceived value of products, alongside recommendations on social media, substantially impacts purchase intentions [34]. Direct interactions on these platforms empower consumers to share their experiences, bolstering their decision-making confidence [35]. Brand engagement promotes a continuous cycle of trust, attitude, and behavior that significantly affects purchasing decisions [36]. Enhanced brand connections, offering benefits such as memberships and discounts, increase the likelihood of consumers favoring the brand in the future.

Brand familiarity and recognition are powerful drivers of purchase decisions [37]. A positive perception of a destination enhances the association between community belonging and the desire to travel [38]. In live shopping environments, consumers’ identification with brands in the broadcast studio significantly boosts the direct effect of interpersonal interactions [39]. In online studio communities, frequent interactions meet community belonging needs. Visible cues such as “add to cart” or “transaction completed” not only inform but also influence other consumers, encouraging compliance and fostering positive behaviors typically culminating in purchases.

The SOBC model is employed to systematically analyze how these elements influence consumer behavior in livestreaming contexts. This model not only assesses the initial stimuli and the organism’s response (consumer feelings and attitudes) but also examines the resulting behaviors (engagement and purchases) and their consequences (brand loyalty and repeat patronage). By integrating the SOBC model, this research aims to delineate the causal relationships between livestreaming stimuli and consumer responses, extending the analysis to the consequences of these behaviors in a restaurant setting. This approach provides a comprehensive framework for understanding how livestreaming can significantly impact consumer behavior, thereby offering valuable insights into enhancing customer engagement and loyalty in the competitive restaurant industry.

Therefore, we hypothesize:

**H4.**  *Social sharing value has a positive impact on behavioral intention*.

Trust is universally recognized as a crucial component contributing to success across all industries, particularly through its dynamic and rational development over time. Studies consistently demonstrate a significant correlation between trust and satisfaction; for example, trust markedly influences customer satisfaction with halal food in Malaysia [40]. Moreover, trust serves as a foundational element in all business partnerships, crucial for ensuring the reliability of transactions [41]. Further research has explored community group buying, highlighting that trust positively impacts customer satisfaction [42].

In the context of restaurant livestreams, trust is cultivated through various visual and interactive cues. Viewers gauge trust based on the cleanliness of the kitchen, the neatness of staff uniforms, and the orderly presentation of fresh ingredients. These elements not only engage and appeal visually but also enhance trust between customers and businesses. A strong brand image and an engaging brand story communicated effectively during these broadcasts significantly bolster this trust, which in turn promotes customer satisfaction [43]. In today’s technology-driven market, trust is central to shaping commercial and trade relationships and influences everything from consumer behavior to business strategies.

This study employs the SOBC model to systematically explore how livestreaming affects key consumer metrics such as trust and satisfaction. By utilizing the SOBC model, this research seeks to delineate the causal relationships between livestreaming stimuli and customer responses, extending the analysis to consider the consequences of these behaviors.

Based on these insights, we hypothesize:

**H5.**  *Trust has a positive impact on satisfaction*.

In the increasingly digital marketplace, where virtual purchasing networks are pervasive, trust has become fundamental to online transactions. Trust in the context of livestreaming encompasses a consumer’s confidence in the accuracy of the information provided, their reliance on the seller’s recommendations, and their assurance that the product delivered will match their expectations [9].

Word of mouth (WOM) in marketing refers to interpersonal communications where consumers share their personal experiences and evaluations of a company or product [44]. Trust is crucial in fostering WOM behaviors, significantly influencing consumer interactions [45]. Electronic word of mouth (eWOM) is recognized by both academics and industry professionals as an influential form of informal communication that impacts relationships between businesses and both current and prospective consumers [46]. A high level of trust in a website or platform significantly enhances travel consumers’ willingness to follow other users’ recommendations and participate in positive word-of-mouth activities [47].

The credibility of product reviews within a community substantially increases the likelihood of these reviews being shared further [46]. In the context of restaurant services, professional livestreaming can establish trust in the quality and service of the establishment. This trust, in turn, encourages customers to share their positive experiences with friends or through social media, thereby generating valuable word of mouth and enhancing the restaurant’s reputation.

Incorporating the SOBC model, this study aims to systematically analyze how livestreaming impacts key consumer metrics such as trust, satisfaction, word of mouth, and behavioral intentions and extends to examine the consequences of these behaviors. By utilizing the SOBC model, we can more comprehensively understand the direct and measurable impacts of livestreaming interactions on consumer behavior, which in turn influences business outcomes. This approach not only assesses the immediate effects of livestreaming stimuli on consumer responses but also explores the long-term consequences, such as enhanced customer loyalty and brand reputation, that stem from increased trust.

Based on these observations, we hypothesize:

**H6.**  *Trust has a positive impact on word of mouth*.

Earlier research has consistently highlighted the critical role of trust in shaping purchase intentions. Within the framework of planned behavior theories, trust is often considered a pivotal explanatory variable influencing consumer decision-making [48]. The connection between brand trust and purchase intentions is well-documented, with studies demonstrating that heightened levels of brand trust significantly enhance the likelihood of purchases [49,50]. This relationship underscores the essential influence of brand trust in boosting consumer purchase behavior.

Furthermore, research exploring customer engagement with livestreams, particularly in contexts such as apparel shopping, has shown that trust plays a fundamental role [51]. Customers are more likely to remain engaged with a livestream if they trust that the apparel being showcased aligns with their expectations and reflects the promised aesthetic. This form of trust is crucial for sustaining interest and engagement in live shopping experiences.

Studies also extend to the dynamics of brand trust and its impact on brand loyalty among restaurant patrons in diverse cultural settings, such as the United States and South Korea [52]. These findings confirm that brand trust acts as a significant determinant of brand loyalty, emphasizing its universal importance across various cultural landscapes.

Based on these insights and the comprehensive framework provided by the SOBC model, we hypothesize:

**H7.**  *Trust has a positive impact on behavioral intention*.

Customer satisfaction has emerged as a pivotal component in marketing, significantly influencing consumer purchasing decisions. It serves as a primary driver of consumer purchases and is a reliable predictor of both loyalty and purchasing intentions [53,54]. The importance of a satisfying consumer experience is critical in determining repeat purchase decisions, with higher customer satisfaction often leading to increased customer loyalty and a greater likelihood of repeat purchases [55,56,57].

Further emphasizing the impact of satisfaction, studies have shown that in the context of fast-food restaurants, the quality of service significantly affects customers’ perceived value, satisfaction, and subsequent behavioral intentions. This underscores that customer satisfaction is a significant determinant of future behaviors, shaping how consumers interact with and return to a business [58].

Given the substantial evidence and the comprehensive approach of the SOBC model, we hypothesize:

**H8.**  *Customer satisfaction has a positive impact on behavioral intention*.

Customer satisfaction stems from perceptions, evaluations, and emotional responses to a product or service experience [59]. It is a crucial factor for marketing success, significantly enhancing a company’s competitive edge [60]. Research has consistently shown that satisfaction with a service or destination significantly boosts the likelihood of engaging in positive word-of-mouth (WOM) activities [61,62]. For instance, customer satisfaction with mobile banking greatly contributes to WOM [63].

Further studies suggest that satisfaction not only drives repurchase intentions but also plays a vital role in generating WOM across different sectors [64]. Users are more likely to disseminate positive WOM following satisfactory experiences [65]. More satisfied travelers, for example, are likely to recommend their destinations [56]. Travelers who enjoy the culinary offerings of a destination are more inclined to share their experiences with peers [54]. Similarly, diners who are pleased with a restaurant’s live food broadcast are likely to endorse it to others. By employing the SOBC model, the research seeks to delineate the causal relationships between the initial consumer reactions to livestreaming (organism) and their subsequent behaviors, such as making a purchase or advocating for the brand (behavior), and the resulting long-term consequences such as increased loyalty or repeat patronage (consequences).

Given these insights, we hypothesize:

**H9.**  *Satisfaction has a positive impact on word of mouth*.

In the realm of restaurant livestreaming, the social exchange theory offers a robust framework for understanding the natural progression from behavioral intention (BI) to word of mouth (WOM) within the SOBC model. This theory, which posits that social behavior is the result of an exchange process aiming to maximize benefits and minimize costs, applies aptly to the digital interactions observed during livestreams. As viewers engage with livestream content, their behavioral intentions—whether to recommend the restaurant, make a purchase, or revisit—are shaped by the perceived value and satisfaction derived from the exchange. These intentions are critical as they lead to WOM, serving as the ‘consequences’ phase in the SOBC model [63].

The linkage between BI and WOM through the social exchange theory suggests that when customers perceive their interactions during the livestream as valuable and rewarding, they are motivated to reciprocate with positive behaviors such as advocating for the restaurant. This reciprocal behavior is driven by feelings of gratitude or loyalty fostered during the livestream, which are typical responses encouraged by the fair and beneficial exchanges outlined by the theory. Thus, the transition from BI to WOM encapsulates the transformation of individual customer intentions into collective endorsements, amplifying the restaurant’s reputation beyond the immediate livestream audience. This pathway not only reinforces the utility of the SOBC model in analyzing digital consumer behavior but also highlights the profound implications of applying social exchange principles to modern marketing strategies in the competitive landscape of restaurant livestreaming [7].

Given these insights, we hypothesize:

**H10.**  *Satisfaction has a positive impact on word of mouth*.

**Figure 1:**
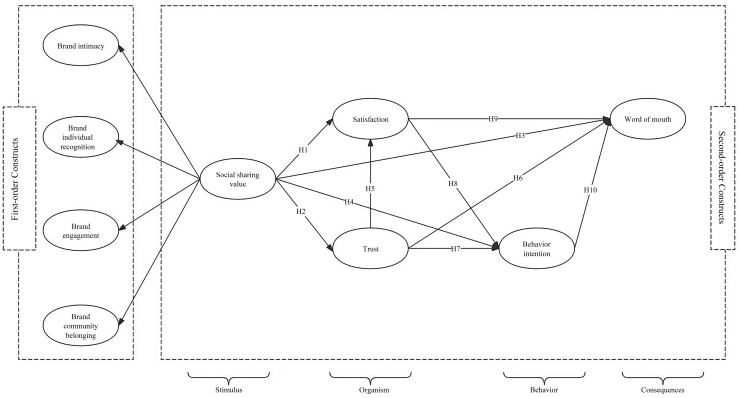
Conceptual model.

Figure 1 describes the research model of this study.

## 3. Research Instruments and Methods

Table 1 offers a comprehensive overview of the measurement and operationalization techniques used in this study, drawing from existing research. This study adopts a quantitative research design utilizing a survey methodology to explore the impact of SSV on customer attitudes and behavioral intentions in the context of restaurant livestreaming. The research employs the SOBC model to systematically analyze these relationships.

The target population for this study includes individuals who regularly view restaurant livestreams in China. A purposive sampling technique was used to ensure the selection of participants who have relevant experience with restaurant livestreaming. This approach helps in obtaining rich and insightful data from respondents who can provide informed opinions about their livestreaming experiences.

The questionnaire was developed based on established measures from existing literature to ensure validity and reliability. It was initially created in English and then translated into Chinese. A pilot test was conducted with a small group of bilingual participants to identify and correct any translation issues and to ensure the accuracy of the content of the questionnaire. Feedback from the pilot test was used to refine the translation and improve the clarity of the questions.

After finalizing the questionnaire, the Chinese version was distributed from 2 August 2023 to 31 August 2023. The distribution was carried out through WeChat groups organized by popular online platforms Meituan and Ele.me, which are well-known for their extensive user base and engagement in the food and beverage sector. Participants of the pilot study were explicitly excluded from the main survey to prevent any bias in the data.

The survey was introduced with a brief explanation of the study’s purpose and instructions for completion. Participants were prompted to reflect on their most recent experience with a restaurant livestream, ensuring that their responses were based on fresh and relevant experiences. The questionnaire included mandatory fields to ensure a 100% completion rate, covering various aspects of SSV, trust, satisfaction, word of mouth, and behavioral intentions.

The initial section of the questionnaire was designed to introduce respondents to the topic of gastronomy livestreaming. It prompted participants to reflect on their most recent experience with a food-related livestream, setting the context for the subsequent questions. To ensure comprehensive data collection, all survey questions were mandatory, resulting in a 100% completion rate from 1139 respondents, as detailed in Table 2. This approach facilitated the collection of robust data essential for analyzing consumer behavior in the context of gastronomy livestreaming.

## 4. Data Analysis

In this study, we employed partial least squares path modeling (PLS-PM) to thoroughly evaluate the predictive validity of our proposed hypotheses through a structured two-stage process.

### 4.1. Evaluation of the Measurement Model

Our examination commenced with a thorough check for common method variance. Given the anonymous nature of the survey, the analysis indicated no significant issues with the method variance, confirming the reliability of the collected responses. To further validate the robustness of our data, Harman’s one-factor test was conducted and revealed that no single factor was dominant, with the largest factor accounting for less than 50% of the variance [70].

In terms of reliability, all composite reliability (CR) values comfortably exceeded the threshold of 0.7, as displayed in Table 1, affirming the internal consistency of our measures. The average variance extracted (AVE) was used to assess the convergent validity of the constructs, with all values surpassing the acceptable level of 0.6. This was further supported by outer loadings and indicator reliability scores, most of which exceeded the 0.7 mark, thereby validating the measurement constructs within our model.

To evaluate discriminant validity, we employed the heterotrait-monotrait ratio (HTMT) [71] developed by Ringle and Sarstedt. The highest HTMT value observed was 0.8918, as shown in Table 3, which falls below the critical threshold of 0.9. This indicates satisfactory discriminant validity across all measured constructs [71].

A disjoint two-stage approach was employed to assess SSV, which comprised four first-order constructs: brand intimacy, brand individual recognition, brand engagement, and brand community belonging [72,73]. Running a redundancy analysis, the global single-item measure of SSV yielded a path coefficient of 0.862 (Convergent Validity), indicating that the four first-order constructs explained more than 50% of the criterion construct variance. Furthermore, the highest VIF value for the four first-order constructs was 2.359, well below the threshold of 5, indicating no significant collinearity issues. The four first-order constructs of SSV achieved weight values of 0.232 (*p* < 0.01), 0.028 (*p* = 0.509), 0.458 (*p* < 0.01), and 0.441 (*p* < 0.01). Although one indicator weight was not significant, the loadings of all four first-order constructs were higher than 0.5. Therefore, the SSV was reliably formed by these four dimensions.

### 4.2. PLSpredict for Model Assessment

The predictive capability of our model was further scrutinized using the PLSPredict procedure, with a particular focus on the Q2 predict statistic values [72]. All values recorded were positive, thereby supporting the predictive relevance of our model. During the PLSPredict analysis, it was observed that the RMSE values for the impulsive consumption indicators were lower than those produced by a corresponding linear model. This result demonstrates a superior predictive accuracy for approximately half of the indicators assessed, with detailed results presented in Table 4.

### 4.3. Evaluation of the Structural Model

Our evaluation of the structural model revealed no significant collinearity issues, with the highest recorded inner VIF value being 2.1857, well below the threshold of 5 [73]. The path coefficients, detailed in Table 5, all achieved statistical significance, supporting the predictive validity of our model across various social sharing value constructs, including brand intimacy, brand individual recognition, brand engagement, and brand community belonging.

The results from our hypothesis testing provide several insights into how SSV and trust influence key consumer metrics in the context of restaurant livestreaming. The strong positive impact of SSV on customer satisfaction indicates that when customers perceive high levels of social sharing value, they are more likely to feel satisfied with the restaurant’s offerings (H1). Elements such as brand intimacy, engagement, individual recognition, and community belonging contribute significantly to this satisfaction. This suggests that restaurants should focus on enhancing these aspects during livestreams to improve customer satisfaction. SSV’s substantial positive influence on trust highlights the importance of fostering a sense of intimacy, engagement, and community during livestreams (H2). Customers who feel recognized and engaged are more likely to trust the brand. This trust is crucial for establishing long-term relationships and loyalty, emphasizing the need for restaurants to cultivate these values in their digital interactions. The positive relationship between SSV and word of mouth (WOM) suggests that customers who perceive high social sharing value are more likely to recommend the restaurant to others (H3). This can be attributed to the enhanced satisfaction and trust derived from the social sharing experience. Restaurants can leverage this by encouraging customers to share their positive experiences, thus expanding their reach and reputation through WOM. The direct effect of SSV on behavioral intention indicates that customers who value their social interactions with the brand are more likely to intend to make a purchase or visit the restaurant (H4). This underscores the importance of creating engaging and valuable livestream content that resonates with customers, encouraging them to take action. Trust’s positive impact on satisfaction reaffirms that customers who trust a brand are more likely to be satisfied with their experiences (H5). Trust can be built through consistent, reliable interactions during livestreams, such as transparency in food preparation and genuine engagement with viewers. This trust, in turn, enhances overall satisfaction with the restaurant. The significant influence of trust on WOM indicates that trusted brands are more likely to be recommended by customers (H6). When customers trust a restaurant, they feel confident sharing their positive experiences with others, which can help attract new customers and enhance the restaurant’s reputation. Trust’s strong positive effect on behavioral intention suggests that customers who trust the restaurant are more inclined to engage in behaviors beneficial to the restaurant, such as making a purchase or visiting the establishment (H7). Building trust through effective livestreams can thus directly drive sales and customer loyalty. The relationship between satisfaction and behavioral intention highlights that satisfied customers are more likely to act in favor of the restaurant, whether through repeat visits or purchases (H8). Ensuring high levels of customer satisfaction through engaging and valuable livestream content can, therefore, translate into tangible business outcomes. Satisfaction’s positive influence on WOM confirms that satisfied customers are more likely to share their positive experiences with others (H9). This finding underscores the importance of focusing on customer satisfaction as a means to drive organic, positive word-of-mouth marketing.

A two-stage approach (Table 6) highlights that brand intimacy (outer loading = 0.9995) is the most vital component of social media sharing value, followed closely by brand individual recognition (outer loading = 0.6937). This emphasizes that emotional attachment—comprising cognitive, affective, and conative aspects—is essential for strong brand relationships. In marketing contexts, consumers who feel a deep attachment to a brand are more likely to dedicate their time, effort, and financial resources to it. This attachment fosters higher-level behavioral responses such as premium purchases, patience with stockouts, spreading positive word of mouth, and active participation in the brand community.

## 5. Discussion

### 5.1. Conclusions

This study focuses on the impact of livestreaming within the restaurant industry, specifically examining how SSV influences customer trust, satisfaction, word of mouth, and behavioral intentions, utilizing the SOBC model. Livestreams serve as the stimulus that triggers a series of consumer responses—both psychological and behavioral—highlighting the dynamic interplay between digital interaction and customer engagement.

Our empirical findings indicate that SSV significantly enhances trust, satisfaction, word of mouth, and behavioral intentions among restaurant livestream viewers. Notably, the impact on trust (path coefficient = 0.5768, f2 = 0.5199) is particularly significant, underscoring the importance for businesses to focus on building trust through their digital strategies. This is aligned with the principles of social exchange theory, which suggests that the development of trust facilitates the progression from transactional to relational exchanges, supporting the results observed in this study [19,35].

The analysis also reveals that brand intimacy (outer loading = 0.9995) stands out as the most influential element of SSV, followed by brand individual recognition (outer loading = 0.6937). This finding emphasizes the crucial role of emotional connections—encompassing cognitive, affective, and conative traits—in establishing strong brand relationships. Consumers with a deep sense of brand attachment are more likely to engage in behaviors that benefit the brand, such as premium purchasing, enduring stockouts, spreading positive word of mouth, and actively participating in brand communities.

Historically, SSV has been structured around four key aspects: brand intimacy, brand individual recognition, brand engagement, and brand community belonging, based on the foundational principles of the social exchange theory as proposed by Homans [10]. This theory emphasizes the optimization of benefits and minimization of costs in social interactions, with fairness playing a crucial role. Prior studies have often not fully captured the dynamic nature of these interactions in digital settings, particularly in livestreaming environments.

In the context of livestreaming, hosts engage viewers through direct and interactive methods such as sharing, displaying, and chatting, which not only effectively transmit business information but also foster a reciprocal relationship characterized by trust, satisfaction, and positive recommendations [13,14]. This aligns with the observed significance of trust as a major influencer of consumer behavior in digital platforms.

The application of the stimulus-organism-behavior-consequences (SOBC) model in this study allows for a comprehensive examination of the customer journey in the context of restaurant livestreaming. By focusing on the progression from behavioral intention (BI) to word of mouth (WOM), the model elucidates how initial interactions during livestreaming sessions (stimuli) impact customers’ intentions (organism responses) and how these intentions lead to observable behaviors such as WOM (behavior), which subsequently result in wider brand reach and reputation consequences.

In particular, the pathway from BI to WOM in the SOBC framework illustrates how strong intentions to engage with or purchase from a brand lead to active advocacy and promotion among peers. This behavioral outcome underscores the significance of livestreaming as a powerful tool for cultivating direct and compelling consumer interactions that not only satisfy immediate engagement metrics but also contribute to long-term brand loyalty and advocacy. The model’s emphasis on consequences helps highlight the strategic importance of managing customer relationships beyond the point of sale, focusing on nurturing ongoing engagement that drives WOM.

Integrating the SOBC model offers valuable insights into optimizing digital marketing strategies within the restaurant industry, emphasizing the need to leverage livestreaming effectively to maximize both immediate engagement and sustained customer relationships. This approach not only addresses a crucial gap in the existing literature on digital consumer behavior but also provides a strategic blueprint for restaurant marketers aiming to enhance both customer satisfaction and brand loyalty through innovative livestreaming tactics.

### 5.2. Theoretical Contributions

This research significantly advances the theoretical understanding of digital marketing within the restaurant industry, particularly through the lens of social exchange theory, by integrating the concept of SSV. Traditionally, social exchange theory has focused on the dynamics of reciprocity and the exchange of resources such as information, goods, and emotional support, emphasizing the strategic calculations individuals make to maximize benefits and minimize costs in their interactions. However, prior studies have often overlooked the intricate dynamics of how digital platforms transform these exchanges, particularly through livestreaming.

The introduction of SSV as a framework in this study expands the traditional scope of the social exchange theory by assessing not just the tangible exchanges but also the depth and quality of interactions that occur in digital environments. SSV, comprised of elements such as brand intimacy, engagement, individual recognition, and community belonging, provides a multifaceted view of how modern consumers interact with brands in real-time, interactive settings. This approach allows for a detailed examination of how these interactions influence consumer behavior, going beyond the simple exchange of goods or services for monetary value.

By applying SSV within the context of livestreaming, the study uncovers how real-time, interactive brand engagements enhance consumer perceptions and behaviors, such as trust, satisfaction, and loyalty. These findings illustrate that digital interactions can foster deeper emotional connections and commitment to a brand, which are crucial for developing long-term customer relationships in the increasingly competitive digital marketplace.

Furthermore, this research contributes to the empirical application of social exchange theory in digital marketing by demonstrating how the qualitative aspects of brand interactions—mediated through livestreaming—affect consumer responses. This not only enriches the theoretical discourse by providing a contemporary application of social exchange principles but also offers practical insights into designing more effective digital marketing strategies that leverage the unique capabilities of livestreaming to enhance customer engagement and brand loyalty.

Moreover, the study explores the specific mechanisms through which SSV operates within the digital marketing landscape. By dissecting the components of SSV, such as brand intimacy, it becomes clear that consumers value personalized and emotionally resonant interactions. This personalization can manifest in various ways during livestreaming sessions, such as direct communication between brand representatives and consumers, real-time responses to consumer queries, and the creation of a sense of community among viewers. These interactions not only satisfy the immediate informational and emotional needs of consumers but also build a long-term sense of loyalty and attachment to the brand.

Engagement, another critical element of SSV, highlights the active participation of consumers in the brand experience. Livestreaming provides a unique platform for such engagement, allowing consumers to partake in product demonstrations, live Q&A sessions, and even virtual events hosted by the brand. This active participation fosters a deeper connection between the consumer and the brand, transforming passive viewers into active participants and advocates.

Individual recognition within the framework of SSV emphasizes the importance of acknowledging and valuing each consumer’s presence and contributions during livestreaming sessions. Personalized shout-outs, responses to individual comments, and tailored recommendations make consumers feel seen and appreciated, thereby enhancing their overall brand experience and reinforcing their loyalty.

Community belonging, the final component of SSV, underscores the collective experience of consumers as part of a larger community of brand enthusiasts. Livestreaming sessions often cultivate a sense of camaraderie among participants, as they share common interests and experiences related to the brand. This sense of belonging not only strengthens individual consumer relationships with the brand but also fosters a loyal and engaged brand community.

However, as livestreaming in the restaurant industry evolves, it could lead to information overload due to the diversity of platforms and the varied nature of livestream content. This overload can overwhelm customers, leading to a sense of fatigue. Nonetheless, empirical findings suggest that effectively managing this overflow of information to highlight relevant content can foster brand intimacy and positively align customer perceptions with the brand ethos. By transforming potential information overload into valuable social sharing, restaurants can create a more engaged and loyal customer base, resonating well with their expectations and preferences.

In conclusion, this research provides a comprehensive framework for understanding the nuanced dynamics of digital marketing within the restaurant industry through the integration of SSV and social exchange theory. By highlighting the importance of brand intimacy, engagement, individual recognition, and community belonging in the context of livestreaming, the study offers valuable insights for marketers aiming to enhance consumer engagement and build lasting brand loyalty. The findings underscore the transformative potential of digital platforms in reshaping consumer-brand interactions, paving the way for more innovative and effective digital marketing strategies.

### 5.3. Practical Implications

First and foremost, social sharing value (SSV) is a critical aspect that restaurant livestreams should prioritize. Our hypothesis testing results strongly support the significant impact of SSV on key consumer metrics. Specifically, SSV positively influences customer trust (H2, path coefficient = 0.5768, *p* < 0.001), satisfaction (H1, path coefficient = 0.4105, *p* < 0.001), word of mouth (H3, path coefficient = 0.2524, *p* < 0.001), and behavioral intentions (H4, path coefficient = 0.2559, *p* < 0.001). During live broadcasts, customers derive significant value from their interactive experiences, fostering trust and generating positive word of mouth, which can drive purchases. Therefore, restaurants need to strategically focus on enhancing SSV during livestreams by increasing viewer participation and creating a sense of belonging to the brand. This can be achieved by blending online and offline experiences and organizing engaging brand activities that provide valuable content. Efficient use of viewers’ time is essential, which can be facilitated by listening to user feedback and ensuring direct communication with customers. Techniques such as modifying account nicknames and prominently displaying the schedule of livestreams in user profiles can help customers easily access broadcast times. Additionally, pre-broadcast promotions through short videos or cross-platform advertising can draw attention to the livestreams. Maintaining a consistent broadcasting schedule, ideally two to three times per day or week at regular times will help cultivate a regular audience.

Brand intimacy is paramount in maximizing the value of social media sharing. Our findings indicate that brand intimacy (outer loading = 0.9995) is the most crucial element of SSV, underscoring its importance in fostering deep emotional connections with the audience. Restaurants should focus on cultivating close relationships with their target audience. Tactics such as incentivizing engagement with “like” button challenges, where participants can win rewards, can significantly boost interaction enthusiasm. During livestreams, offering periodic rewards such as ‘blessing bags’—akin to gamified blind boxes—can keep viewers engaged and motivated. These activities foster viewer loyalty and enhance the interactive dynamics of the broadcast. Engaging in collaborative events with other brands, such as friendly competitions between restaurant livestreams, can further enrich the viewing experience and highlight the restaurant’s unique offerings.

The profound impact of social media sharing value on trust underscores its fundamental role in the restaurant industry. Our results show that trust significantly impacts customer satisfaction (H5, path coefficient = 0.4190, *p* < 0.001) and word of mouth (H6, path coefficient = 0.2599, *p* = 0.0019). To build trust through livestreaming, restaurants could showcase an open kitchen concept, ensuring that cleanliness, dish presentation, and staff professionalism are visibly upheld. Emphasizing food safety by demonstrating advanced traceability systems during broadcasts reassures customers about the quality and origin of the food. Interactive segments showcasing the journey of ingredients from farm to table, including detailed records of food handling practices, can provide transparency. Further, demonstrating the freshness of ingredients, such as using in-house vegetable planters and showing their growth and harvesting live, can visually affirm the restaurant’s commitment to quality and freshness. Engaging well-known personalities or staff in these segments can also draw viewer interest and participation.

From a societal perspective, enhancing SSV through livestreaming can contribute to greater transparency and trust in the food industry, encouraging healthier and safer dining choices among consumers. Methodologically, this study highlights the importance of using advanced digital marketing strategies to foster strong customer relationships. By strategically enhancing these elements, restaurants can leverage livestreaming not just as a marketing tool but as an effective means for cultivating lasting relationships with customers, fostering trust, and creating a vibrant, interactive community around their brand. This aligns with our findings that SSV has a substantial effect on key consumer metrics, reinforcing the need for restaurants to adopt these strategies to achieve sustained success in the digital marketplace [74].

### 5.4. Limitations and Future Research

The current study is geographically limited to China, which may not fully represent global consumer behavior trends. Expanding the research to include diverse cultural contexts, such as Southeast Asia and Western countries, would help generalize the findings and better understand regional variations in livestreaming’s impact on consumer behavior.

Future research could also investigate the impact of livestreaming across different segments of the restaurant industry, such as fast food versus fine dining. Understanding how different types of restaurants leverage livestreaming could offer insights into tailored strategies that maximize consumer engagement and satisfaction based on the specific characteristics and expectations of each segment.

Furthermore, longitudinal studies could provide a deeper understanding of the long-term effects of livestreaming on consumer behavior and brand loyalty. Tracking changes in consumer attitudes and behaviors over time in response to continued exposure to livestream marketing could help identify trends, shifts in consumer preferences, and the durability of livestreaming impacts.

Another potential area of expansion could involve the integration of machine learning and artificial intelligence in analyzing livestreaming data. These technologies could help in automating the recognition of patterns in consumer interactions and feedback during livestream sessions, thereby enhancing the precision of consumer insights and allowing for real-time adjustments to marketing strategies.

Moreover, it would be beneficial to examine the psychological drivers behind consumer engagement in livestreaming, such as the role of social presence, parasocial interaction, and entertainment value. Such psychological insights could aid marketers in designing more compelling livestream content that resonates emotionally with viewers, fostering a stronger psychological connection and potentially leading to higher conversion rates.

Lastly, considering the rapid evolution of technology and consumer digital habits, investigating emerging trends and innovations in livestreaming technology—such as virtual reality (VR) and augmented reality (AR) applications—could provide valuable foresights into the next wave of digital marketing strategies. These technologies could transform the livestreaming experience from a viewing activity into an immersive interaction, creating new opportunities for engaging consumers in novel and memorable ways. This research would not only fill existing gaps but also advance theoretical and practical knowledge in the field of digital marketing within the hospitality industry.

## Figures and Tables

**Table 1:** Measurement and operationalization.

Construct	Items	Loading	Composite Reliability	AVE
Social sharing value		-	-	-
	Brand intimacy [66]			
	I experience a form of connection between this brand and me.	0.7576		
	I feel closer to this brand.	0.7169		
	I feel there is more intimacy between this brand and me.	0.7562		
	Brand individual recognition [66]			
	I feel I’m better recognized as a customer by the brand.	0.7436		
	I feel I’m treated with more consideration by this brand.	0.7329		
	I feel I am treated with more regard by this brand.	0.5788		
	Brand engagement [67]			
	I can influence the brand and its products.	0.5827		
	I can help improve the brand and its products.	0.6327		
	I feel that my comments and suggestions will influence the brand and its products.	0.8208		
	Brand community belonging [66]			
	I feel a sort of connection with others who use this brand.	0.6850		
	I feel connected to people who share the same interests as me.	0.6806		
	I feel close to people who share the same views about this brand.	0.7074		
Satisfaction [68]			0.8858	0.6597
	I am satisfied with my overall experience with this brand.	0.8141		
	I believe that choosing to shop at this supermarket was the right decision.	0.8218		
	My interactions with this brand meet my expectations for this type of brand.	0.8109		
	I would be very happy to purchase from this brand again.	0.8019		
Trust [69]			0.8855	0.6592
	I believe Restaurant A strives to keep its promises to customers.	0.8208		
	I would like Restaurant A to continue providing quality services.	0.8237		
	I feel confident in Restaurant A.	0.8145		
	Restaurant A never disappoints me.	0.7882		
Word of mouth [64]			0.8795	0.6459
	I say positive things about Restaurant A to other people.	0.7972		
	I recommend Restaurant A to someone who seeks my advice.	0.8034		
	I am likely to encourage others to consider Restaurant A.	0.8161		
	I am likely to recommend Restaurant A to my friends or acquaintances.	0.7980		
Behavior intention			0.8684	0.6233
	It is likely that I will repurchase from Restaurant A in the near future.	0.7978		
	In the future, I intend to continue using services from Restaurant A.	0.8133		
	I shall continue considering Restaurant A as my main restaurant in the coming years.	0.8249		
	I would remain a customer of Restaurant A even if another restaurant offered better rates.	0.7173		

**Table 2:** Respondent profile.

Measure	Item	Frequency	(%)
Gender	Male	559	49
	Female	580	51
Age	<18	18	1.5
	18–25	244	21.4
	26–30	358	31.4
	31–40	330	28.9
	41–50	128	11.2
	51–60	54	4.7
	>60	7	0.6
Education	Junior high school or below	46	4
	Senior high	124	10.8
	University	888	77.9
	Master’s degree or above	81	7.1
Income	<1000	63	5.5
	1000–2000	72	6.3
	2000–3000	93	8.1
	3000–5000	278	24.4
	5000–10,000	450	39.5
	>10,000	183	16

**Table 3:** Heterotrait-monotrait (HTMT) ratio of correlations and Fornell–Larcker criterion.

	Behavior Intention	SSV	Satisfaction	Trust	Word of Mouth
Behavior intention	0.7895	-	*0.8178*	*0.7969*	*0.8918*
SSV	0.6311	-	-	-	-
Satisfaction	0.6673	0.6556	0.8122	*0.7914*	*0.741*
Trust	0.6475	0.5849	0.6558	0.8119	*0.7111*
Word of mouth	0.721	0.5899	0.6099	0.5857	0.8037

Notes: The HTMT result is highlighted in italics and falls above the diagonal value, while the below result belongs to Fornell–Larcker Criterion.

**Table 4:** PLSpredict assessment on manifest variables.

	Q^2^_Predict	PLS-RMSE	LM RMSE	PLS-SEM–LM RMSE
WOM1	0.213	1.051	1.053	−0.002
WOM 2	0.21	1.021	1.022	−0.001
WOM 3	0.261	1.042	1.042	0
WOM 4	0.176	1.056	1.057	−0.001

Notes: RMSE = root mean squared error; PLS = partial least squares path model; LM = linear regression model; k = 7 subgroups, number of repetitions = 10.

**Table 5:** Structural model results—effects on dependent variables.

	Direct Effect	*p*-Value	Bootstrap Confidence Interval		f Square	Supported
Behavior intention ($R^{2}$ = 0.5572 $Q_{predict}^{2}$ = 0.3881)						
Social sharing value (H4)	0.2539	0.0008	0.0473	0.1328	0.0799	YES
Trust (H7)	0.3016	0.0003	0.0646	0.1651	0.1060	YES
Satisfaction (H9)	0.3044	0.0001	0.0519	0.1370	0.0945	YES
Word of mouth ($R^{2}$ = 0.5683 $Q_{predict}^{2}$ = 0.3387)						
Social sharing value (H3)	0.1292	0.0003	0.0421	0.1105	0.0695	YES
Trust (H6)	0.1153	0.0019	0.0360	0.1052	0.0642	YES
Satisfaction (H8)	0.1309	0.0013	0.0317	0.0969	0.0629	YES
Behavior intention (H10)	0.4792	0.0001	0.4171	0.5362	0.2353	YES
Satisfaction ($R^{2}$ $=0.5426\;Q_{predict}^{2}$ = 0.4208)						
Social sharing value (H1)	0.4105	0.0000	0.1844	0.3411	0.2460	YES
Trust (H5)	0.4190	0.0000	0.1619	0.3372	0.2465	YES
Trust ($R^{2}$ = 0.3425 $Q_{predict}^{2}$ = 0.3336)						
Social sharing value (H2)	0.5768	0.0000	0.4041	0.6707	0.5199	YES

**Table 6:** Assessment of higher-order construct on cross-functional cooperation.

High-Order Construct	Low-Order Construct	Outer Loading	Outer Weights	t-Value	CI		VIF	Convergent Validity
Social sharing value	Brand intimacy	0.9995	0.232	1217.4222	0.9973	0.9998	2.3587	0.8623
	Brand individual recognition	0.6937	0.028	28.2241	0.6507	0.7302	2.3517	
	Brand engagement	0.6083	0.458	21.3508	0.5626	0.6579	1.973	
	Brand community belonging	0.568	0.441	18.3599	0.5131	0.6145	1.8328	

Note(s): CI means 95% confidence interval bias corrected.

## Data Availability

The data presented in this study are available on request from the corresponding authors.
